# Perceptions of Cannabis Use: A Qualitative Descriptive Study of Rheumatology Patients

**DOI:** 10.1177/21501319231194974

**Published:** 2023-08-26

**Authors:** Joanne Olson, Hailie Brophy, Tarek Turk, Allyson Jones, Shelby. S. Yamamoto, Elaine Yacyshyn, Cheryl A. Sadowski, Pauline Paul

**Affiliations:** 1 University of Alberta, Edmonton, AB, Canada

**Keywords:** arthritis, cannabis, patient perspectives, rheumatic conditions, qualitative research, rheumatology

## Abstract

**Introduction/Objectives:**

Some rheumatology patients use or contemplate using cannabis, however, may not be transparent about use with their providers. The objective of this qualitative descriptive study was to describe beliefs, perceptions, and learning needs of adults with rheumatic conditions regarding the use of cannabis products.

**Methods:**

Purposive sampling was conducted through a rheumatology clinic and sought participants who were using or thinking about using cannabis. Two online focus groups based on cannabis use patterns (non-users and users) were conducted separately. Interviews were audio recorded and transcribed. Three research team members read the transcripts independently to identify initial codes and themes. Data saturation was reached with the interviews.

**Results:**

We recruited 12 participants between 52 and 85 years old. The first theme was pain and desperation. Stigma was the second theme with a perception of physician opposition to cannabis, and the reluctance of many participants to discuss cannabis use with physicians. The final theme was a need for information and a general lack of trustworthy and credible sources. Users were willing to try cannabis even if they still had questions.

**Conclusion:**

Rheumatology patients are open to using cannabis due to the burden and suffering associated with pain. They remain silent on the topic, however, because of stigma and lack of engagement from health care professionals, particularly physicians. Patients voiced a strong need for information regarding cannabis and want healthcare providers to initiate discussion. These findings are clinically relevant to the management of rheumatic conditions and the promotion of therapeutic relationships.

## Introduction

Individuals with rheumatic conditions frequently report that current treatment strategies do not lead to effective pain control.^1-3^ It is known that many people living with chronic pain consider cannabis as an option. In 2001, Canada regulated medical cannabis use. Since 2018, it is legal to sell cannabis in retail outlets.

Pain is the most common reason to use medical cannabis.^4-7^ Older individuals are increasingly turning to cannabis to deal with chronic pain and other issues such as insomnia.^8^ Yet there is limited evidence about the effects of cannabis for people living with rheumatic conditions.^1^ Therefore, both clinicians and individuals living with rheumatic conditions face uncertainties and unanswered questions.

The overall aim of this study was to develop a tool to guide clinicians and rheumatic patients about cannabis use. The World Health Organization^9^ recommends that qualitative findings be part of the process of developing guidelines. In this paper we present such findings.

The objective of the descriptive qualitative component of the study was to uncover beliefs, perceptions, and learning needs of adults with rheumatic conditions regarding cannabis. We suspected that patients hesitate to share their views with care providers. While conducting this study, Kelly et al^10^ confirmed our clinical experience in a publication that recommended qualitative research be conducted with rheumatology patients to expose experiences and perceptions often not shared with clinicians.

## Methods

A qualitative descriptive design was selected since little is known about the topic of interest.^11^ The study took place in a large urban center in Alberta, Canada. After receiving ethics approval, potential participants were recruited through the local academic center rheumatology clinic. The clinicians identified adults with rheumatologic diagnoses and invited them to contact the research assistant for study participation if they were interested. Care providers were not informed about patient decisions to participate or not participate in the study. Patients who agreed to participate provided written consent and were interviewed in focus groups using a semi-structured interview guide developed by the research team. Two focus groups based on cannabis use patterns (users and non-users) were conducted separately.

Data collection occurred during the COVID-19 pandemic with focus groups taking place virtually using the Zoom platform. Two team members were present for each 1-h focus group as it is standard practice in this data collection approach.^12^ One researcher facilitated the discussion while the other observed and insured that all interview questions were addressed. Data were transcribed verbatim. In order to ensure rigor, 3 team members independently conducted initial coding and theme identification. Finally, they discussed their individual analysis and reached consensus on the final themes presented here.

## Results

A total of 12 individuals participated in the study. Fifty-eight percent were in their 50s while 42% were 60 or older. In the user group (n = 7) the mean age was 62 years (range 53-85); 5 participants identified as female and 2 as male. In the non-user group (n = 5) the mean age was 60 years (range 52-73); 4 identified as female and 1 as male. Definitions of older persons vary with the start of older age from 50 or 60 years and over. We decided to identify all participants as members of the older population. We did not seek information about specific diagnoses as we were interested in experiences with and views about cannabis. Although there is limited consensus on sample size for qualitative research, data saturation is the norm for determining sample size.^13^ We are confident that data saturation occurred, since participants in both groups shared very similar views. Here we present the 3 themes that emerged from the analysis: pain and desperation; stigma; and need for information. Table 1 provides representative quotes for each theme among cannabis users and non-users.

**Table 1.** Supporting Quotes by Themes Among Cannabis Users and Non-Users.

Theme	Cannabis users	Cannabis non-users
Pain and desperation	“Yeah, I don’t think there’s other ways. It just helped me with that, with pain. And all pain. Not – not just arthritis.” (Participant user 1) “I think it helps mostly with the pain. Then the decreased pain allows you to get more mobile. If I do a lot of work and overuse my hands, they really swell. I ice them and things like that, but it doesn’t always relieve them completely [So I use cannabis].” (Participant user 2) “You sleep like a baby, and you don’t hurt.” (Participant user 3) “I used to love to walk, and I can’t anymore. But other than that, it’s made things tolerable. But now I can, you know, I can sit in my car and drive a fairly long distance without too much pain.” (Participant user 5*)* “Last night, I increased my oil by another mL, so I actually slept an extra hour. So, I take it as a win. Because I’m using it, it helps me being able to have more of a quality of life, and that is very important to me.” (Participant user 7).	“I’m really interested in [cannabis] because sometimes the pain is really bad.” (Participant non-user 5) “. . .it’s everybody’s choice, I don’t want to use it now. I’m not in pain to the extent that I feel I want it.” (Participant non-user 1) “Pain management is the new “buzzword” right? It’s all about pain management but they don’t give you all the tools to manage pain. You can ice, use Tylenol [acetaminophen], elevate, but if it doesn’t work, what do you do? We’re all trying to come up with something, with anything that is going to help.” (Participant non-user 4) “You know, it’s just to relieve some pain. Once the pain is relieved, you’re fine. You know, you’ve already got it covered.” (Participant non-user 2). “I think the side effects from cannabis are much less than the side effects of some of the arthritis drugs that I’ve been on. So. . . I’m not worried about that. I’m more worried it won’t do anything for me and then, again, that’s another avenue I’ve explored, and there’s been no result. Because I’ve tried everything.” (Participant non-user 4)
Stigma	“I think about it as a generational thing, because when we grew up, the bad kids did the weed. I am nervous walking in a cannabis store . . .thinking, what if someone sees me and goes, ‘Oh, . . ., I saw her going into a weed store.’ This concerns me. There is that stigma, you know. Even though I find if you tell anyone you’ve tried it, they’ve tried it as well.” (Participant user 6) “It’s one of those things that, you know, if the doctor doesn’t suggest it to you, you’re not going to bring it up. Because yeah, people go, “Oh, you know, you’re just like a drug addict.” (Participant user 2*)* “I didn’t want to bug [my physician] because, you know, there’s stigma attached to it, and I love [my physician]. [Laughs] . . . I think it carries that stigma with it, so it’s hard to talk to my doctor about it, because I feel like okay, that’s something that I can deal with [on my own].” “Nurses and doctors seem to be fine with it. There seems to be a new attitude. I think the biggest thing is, it's now legal.” (Participant user 3) “You are kind of really caught, then, aren’t you? Because if you can’t talk to your doctor about it, if you’re not comfortable doing that, and [cannabis store employees] can’t give you any advice, where do you go for advice?” (Participant user 1)	“If I could take a gummy and feel relief, I would [take it]. But I don’t want to sit on the deck and have someone say [sniffing] ‘what’s that smell? It goes back to high school when there were the kids that did drugs and I was the ‘good girl’. . . It doesn’t sit right with me. . . just can’t get past that.” (Participant non-user 5) “Part of our fear is that sometimes people start with marijuana and then that’s not enough and they start using heavier drugs.” (Participant non-user 1) “I have the same stigma thing. I wouldn’t want [to go to a store]. I still would rather it come from a pharmacy.” (Participant non-user 1) “I am 52 and I still have problems walking into a liquor store because there’s all that, you know, stigma around it.” (Participant non-user 3) “Doctors and lawyers are doing it and. . . I think they’re pretty intelligent people.” (Participant non-user 2) “I’ve got people in my family who smoke pot like it’s nothing, right?” (Participant non-user 1) “There’s a lot of people waiting in line to get those doctors. If they get peeved at you, you’re going to be in a predicament.” (Participant non-user 5) “I wonder what would happen if we tried cannabis, felt terrific, and then all of sudden they’re saying ‘you don’t need such and such [medications] anymore’. That could be a real issue for us to have medications taken away.” (Participant non-user 4)
Need for information	“My daughter suggested that I try it. . . She had been in stores and had seen a sign that said “pain management.” (Participant user 2) “One of my coworkers had a very bad back, and he came in espousing the wonders of CBD.” “I talked to my GP, and he doesn’t prescribe marijuana, so he recommended another doctor. This doctor . . . was extensive in his questioning, and looked at my medical background, and we determined it should be something that works.” (Participant user 5) “I went to a cannabis store [and they] were not helpful at all. . . because they said, ‘I’m not a doctor. You tell me what you want’.” (Participant user 4) “I think [cannabis store employees] are not allowed to talk about therapeutic elements because I think all the licenses are recreational. I asked at one store about pain management, and they said, ‘We can’t even talk about it, because we could lose our license’. “[It would be helpful if] there was a portal where people could go and find information that would be physician managed. ”Participant user 3) “I think the primary care networks that are here in the city. . .there’s no reason in the world why they couldn’t do it.” (Participant user 1) “If the physician said ‘Have you ever considered using this in pain management?’ You know, then it opens the door. I took [a course] offered by a physio and it was on icing, and other ways to manage pain. But again, nothing is ever [said about cannabis].” (Participant user 2)	“I have no idea about side effects. I need more information. How much do I take? What do I take?” (Participant non-user 1) “[I need information about] whether you get addicted, or what to take and how to use it.” (Participant non-user 5) “I’m interested, I’ve watched a lot of things on YouTube and 60 Minutes and people from [some other countries] use cannabis . . . to get over their pain.” (Participant non-user 2) “I would feel comfortable if [information] was coming from doctors. They have pamphlets on breast feeding and osteoporosis and. . . I would like some for [cannabis]. And then be able to discuss with them, I’d feel more comfortable.” (Participant non-user 1) “That’s why I would prefer a medical professional. . . This is like medication. Start with this, oh that’s too much, that’s too little. There’s a lot of misinformation out there. . . it should be under [our provincial health authority]so that people know the information has been researched and it’s not just hearsay. This is what the facts are and these are the pros, and these are the cons.” (Participant non-user 5) “It would be nice to talk to your pharmacist. . ..” (Participant non-user 4) “I think any health professional would be good to talk with. Definitely nurses, they know a lot, doctors, and pharmacists.” (Participant non-user 3) "I think it would be great to have a health professional, whether it was like a guest speaker or a pop-in. I would like a cannabis specific group.” (Participant non-user 1) “It would be nice to find a support group just of normal people that have arthritis and would share their experiences. I would sign up in a heartbeat.” (Participant non-user 3)

### Pain and Desperation

Pain was a trigger to either use cannabis products or seek more information about potentially using these products. Users initiated cannabis consumption in response to pain and having a desire to improve their quality of life. Pain was closely connected to mobility and swelling, with participants discussing other methods that may not have worked for them to control these symptoms. All participants who used cannabis products found them helpful in promoting restful sleep with less pain.

Non-users shared that pain is not well controlled and considered that pain management would justify turning to cannabis. However, as the discussion on pain continued, it became clear that there was a level of desperation in most of the participants as they tried to find solutions to living with their pain. This desperation was reflected in statements made during the focus group interviews.

### Stigma

Both users and non-users addressed stigma associated with cannabis. Entering a cannabis retail store was an issue for some users. Stigma also made them reluctant to talk about their cannabis practices with their care providers. Participants who used cannabis were not smoking it. Other forms of cannabis, including oils and edibles, were more socially acceptable for participants. For some users, the legalization of cannabis had removed the associated stigma, while for others it remained.

Non-users wanted to know more about cannabis products but were generally also opposed to smoking cannabis. Hesitation to use cannabis seemed related to the societal norms of their youth, including the common concern that cannabis can lead to other drugs. Non-users expressed discomfort entering cannabis retail outlets that was similar to feelings they had about being in a liquor store. However, non-users were beginning to be reassured, knowing that some professionals and family members were consuming cannabis.

Stigma contributed to hesitancy in talking with care providers as it could potentially alter relationships. Users preferred when physicians introduced the topic of cannabis use. Non-user participants considered their relationships with care providers to be critical in the management of their health conditions and not something they were willing to compromise. They feared that cannabis use or discussing it would negatively impact relationships with health professionals as well as subsequent treatment of their condition.

### Need for Information

Both users and non-users were seeking information about cannabis from various sources. Users relied on family members, friends, cannabis stores, and some health professionals for information. Accessing information was often challenging. Non-users expressed many learning needs about cannabis and were curious about it. Both users and non-users wanted credible sources of information to be more available. Non-users wanted health professionals to offer specific information about dosage. They also desired that health care organizations would provide information on cannabis on their websites.

Participants wanted health professionals to raise the topic of cannabis use during their routine interactions. Some users were comfortable talking with physicians about their use of cannabis. However, 1 user wished that cannabis had been discussed as an option in formal group sessions on pain management. When further probed on this topic, participants identified many members of the health care team who could provide information, including physicians, pharmacists, nurses, and rehabilitation therapists. Both users and non-users recommended that health professional-led cannabis-use support groups would be beneficial.

## Discussion

This study is among the first to explore the experiences of cannabis users and non-users in a sample of people living with rheumatic conditions. Similar to other studies on a variety of conditions^4-7,14-16^ we learned that pain was the main reason for using or considering the use of cannabis. Smoking cannabis carried a stigma and users preferred other forms including oils and edibles. Other research with older adult users indicates a similar preference.^14^ Although non-users were curious about cannabis, lack of information prevented them from consuming. Even if cannabis use is becoming normalized in Canada and beyond,^17,18^ stigma remains and is a factor in decision making in both younger and older populations.^15,19^

In this study, both users and non-users were hesitant to discuss cannabis use with health care providers. The presence of stigma may have contributed to participants’ desire for health professionals to initiate conversations about cannabis use. In addition, physician opposition to cannabis, whether real or perceived, seemed to generate fears that mentioning cannabis would alter relationships. The risk of harming relationships was considered when deciding to be open or not about using or wanting to try cannabis. Similar to our findings, a survey of 276 cannabis users revealed that 79.3% of participants were hiding their use as they feared physician judgment.^20^ This may lead to a culture of self-reliance, secrecy, and viewing cannabis as an over-the-counter product purchased in retails settings. In these stores, there are no interactions with clinicians therefore there is a risk for incomplete information and even misinformation.

On the other hand, participants trusted health care providers and sought credible evidence about using cannabis for pain management. They wanted these providers to bring up the topic of cannabis use in an open and non-judgmental manner. Furthermore, they sought guidance about benefits and risks of cannabis use as a treatment option for pain. Other research has also found that care providers offer limited guidance.^14,15^ Participants were seeking the integration of cannabis discussions into pain management programs led by a variety of health professions. Overall, users and non-users were similar in wanting information about cannabis. The main difference was that non-users did not want to initiate cannabis consumption without more information, while users were more tolerant of risk and were willing to initiate cannabis even if they had information needs. Of interest a survey of 638 older adults conducted by Active Aging Canada in 2018, indicated that 40% of the participants who did not use cannabis wanted to know more about risks and benefits.^21^

Based on the findings of this study and other studies,^14,15,22,23^ it would be optimal for health care providers to routinely initiate discussions about cannabis and pain management. Research indicates that healthcare providers have divergent views about the use of cannabis.^24^ However, they should be aware that older adult populations may be using it, which could have implications for their health.^25,26^ For, example, in a large Canadian survey it was found that between 2014 and 2019, the number of patients with rheumatic conditions using cannabis tripled (to almost 13%), and users were increasingly older females. Open and honest discussion about this topic could contribute to quality care, patient safety, and better patient outcomes. The health care system needs to be more proactive in the dissemination of evidence-based information about cannabis. There is an opportunity to more systematically provide information in clinics, pharmacies, and through health care agency websites. Findings suggest that research is needed to better understand the views of physicians and other health professionals about cannabis use, and their confidence in approaching the topic with patients. More multi-site research is needed with larger groups of users and non-users.

### Limitations and Strengths

The limitations of our study include recruiting participants at only 1 academic rheumatology clinic and the challenges of engaging potential participants during COVID-19. Nonetheless, the findings were similar to that of other projects^4-7,14-17,19-24^ that involved different patient populations. The strengths of our study included: a broad age range of older adult rheumatology patients was recruited; a balanced representation of cannabis users and non-users; and the fact that this is one of the few studies that specifically examined cannabis use in a rheumatology patient population.

## Conclusion

We consider the most important finding for practice to be that participants wanted health professionals to initiate conversations about cannabis use. They sought this in part because of the lingering stigma even after legalization. For participants, health professionals approaching the subject is destigmatizing and can facilitate discussion about goals related to pain management. It is clear that patients want more information, and an open, nonjudgmental relationship with their providers as they search to manage pain and other symptoms in the context of rheumatic conditions. If health care providers do not initiate discussion about cannabis use, there is a risk that patients with rheumatic conditions will conceal their use which could have negative health implications.
